# Supplementary material for: Investigating pathways of vancomycin-resistant Enterococcus (VRE) contamination and transmission in intensive care units: a prospective genomic surveillance study
Source: medRxiv. 2026 Jul 30:2026.07.28.26359040. Preprint. [Version 1] doi: 10.64898/2026.07.28.26359040 (PMC13484347; doi:10.64898/2026.07.28.26359040)
Supplement: Supplementary file 1 [file NIHPP2026.07.28.26359040v1-supplement-1.pdf]

medRxiv preprint doi: https://doi.org/10.64898/2026.07.28.26359040; this version posted July 30, 2026. The copyright holder for this preprint (which was not certified by peer review) is the author/funder, who has granted medRxiv a license to display the preprint in perpetuity.
It is made available under a CC-BY 4.0 International license .

Supplementary Material

Section 1: Environmental Sampling Methods

Table S-1. Hospital A total surface areas of environmental surfaces and surface composites.

Surface	Description	Total surface area of composite
Patient room Zone 1	Composite of near-patient touch area surfaces – bedrails, over bed table, call button/remote; patient/provider touch overlap areas	194 sq in
Patient room Zone 2	Composite of healthcare personnel touch area surfaces – computer keyboard, scanner handle, room supply cart handles, IV pole high touch area	300.5 sq in
Patient room Zone 3	Composite of patient toileting area surfaces – toilet grab bars, flush handle, rinse spout. Alternatively: commode handles if commode present	76 sq in
Patient over bed table	High touch area of patient over bed table (relevant precautions rooms only)	132 sq in
Patient bedrails	High touch area of both patient bedrails (relevant precautions rooms only)	108 sq in
Door touch areas	High touch areas of door – handles, door grab areas (relevant precautions rooms only)	130 sq in
Sinks	Sink area – sink top, handles, soap dispenser button, hand sanitizer dispenser button	114 sq in
Shared unit equipment	Surfaces of reusable shared unit equipment used in patient rooms	Variable
Shared nursing area	Composite of high touch areas in nurses station – two computer keyboards and two telephone handles	140 sq in

medRxiv preprint doi: https://doi.org/10.64898/2026.07.28.26359040; this version posted July 30, 2026. The copyright holder for this preprint (which was not certified by peer review) is the author/funder, who has granted medRxiv a license to display the preprint in perpetuity.
It is made available under a CC-BY 4.0 International license .

Table S-2. Hospital B total surface areas of environmental surfaces and surface composites.

Surface	Description	Total surface area of composite
Patient room Zone 1	Composite of near-patient touch area surfaces – bedrails, over bed table, call button	173 sq in
Patient room Zone 2	Composite of healthcare personnel touch area surfaces – computer keyboard, scanner handle, in-room medication dispenser, IV pole high touch area	181 sq in
Patient room Zone 3	Composite of patient toileting area surfaces – toilet grab bars, flush handle. More commonly VA patients used commodes. Commode handles sampled if commode present	61 sq in
Patient over bed table	High touch area of patient over bed table (relevant precautions rooms only)	124 sq in
Patient bedrails	High touch area of both patient bedrails (relevant precautions rooms only)	108 sq in
Door touch areas	High touch areas of door – handles, door grab areas (relevant precautions rooms only)	130 sq in
Sinks	Sink area – sink top, handles, soap dispenser button, hand sanitizer dispenser button	87 sq in
Shared unit equipment	Surfaces of reusable shared unit equipment used in patient rooms	Variable
Shared nursing area	Composite of high touch areas in nurses station – two computer keyboards and two telephone handles	140 sq in

Section 2: Microbiologic methods

To specifically isolate presumptive vancomycin-resistant *Enterococcus* strains, methods for sponge samples and swab samples vary slightly, but both involved culturing on VRE Chrome ID agar plates or in Bile Esculin Broth (BEA) with a vancomycin disk. Sponges were processed in a stomacher bag with 45 mL 0.02% Tween 80 buffer and the sponge eluate concentrated by centrifugation. To quantify VRE, 200 μL of each sponge eluate was pipetted onto VRE Chrome ID agar plates. For broth enrichment of the eluate, 500 μL was dispensed into bile esculin azide (BEA) broth with a 30 μg vancomycin disk. For

medRxiv preprint doi: https://doi.org/10.64898/2026.07.28.26359040; this version posted July 30, 2026. The copyright holder for this preprint (which was not certified by peer review) is the author/funder, who has granted medRxiv a license to display the preprint in perpetuity.
It is made available under a CC-BY 4.0 International license .

swab samples, 100 μL of the vortexed swab transport media was plated onto VRE Chrom ID plates and 100 μL dispensed into BEA broth with a 30 μg vancomycin disk.

Plates were checked for growth at 24 and 48 hours, and the corresponding broth checked at 48 hours if no enterococci growth was seen on a plate. Plates and broths were held for 72 hours before discarding. If enterococci growth was observed on VRE Chrome ID plates, colony counts were recorded after 48 hours, and morphologically-distinct suspected enterococci colonies were subcultured to blood agar for identification by MALDI-TOF (Bruker Biotyper CA System, Bellerica, MA). If VRE Chrome ID agar plates were negative for enterococci at 48 hours, but growth was observed in the bile esculin broth with vancomycin disk, broth was streaked onto VRE Chrome ID agar plates for confirmation.

Section 3: Alignment-based genomic methods

Raw reads were trimmed for adapters and contaminants using bbduk from bbmap (version 39.10)[37] and then quality-checked using fastqc (version v0.12.1).[38] We used SPAdes (version v4.2.0)[39] to assemble contig short reads in full and plasmid assemblies. To identify specific resistance and virulence genes (Table S-3), full and plasmid assemblies were annotated using bakta (version 1.12.0)[40] against the full database version 6.0.[41] To identify the clade of each VRE isolate, assemblies were run against reference genomes for each clade A1, A2, and B (Table S-3) using fastANI (version 1.34)[25] to determine the closest match. We used snippy (version 4.6.0)[42] to create a single core alignment of each isolate against their respective reference genomes. To account for recombination, we ran gubbins (version 3.4)[43] to establish a phylogeny of all isolates and then ran snp-dists (version 0.8.2)[44] to establish pairwise single nucleotide variants (SNVs).

Section 4: Genomic clustering sensitivity analysis

All methods were used with both SNV thresholds commonly used in the literature: 7[12] and 20.[9,34] All combinations of methods, parameters, and SNV thresholds were included in the sensitivity analysis (Table S-5). Any combination of methods, parameters, and SNV thresholds that produced different clusters was reported.

medRxiv preprint doi: https://doi.org/10.64898/2026.07.28.26359040; this version posted July 30, 2026. The copyright holder for this preprint (which was not certified by peer review) is the author/funder, who has granted medRxiv a license to display the preprint in perpetuity.
It is made available under a CC-BY 4.0 International license .

Table S-3. Key *Enterococcus* resistance and virulence factor genes

Category	Gene	Function	Source
Resistance	*vanA, (vanS-A, vanR-A, vanY-A, vanX-A, vanH-A)*	Vancomycin resistance	[45]
Resistance	*vanB*	Vancomycin resistance	[45]
Resistance	*vanZ*	Teicoplanin resistance	[45]
Virulence factor	*ace/acm*	Collagen adhesion	[46]
Virulence factor	*asa1*	Adhesion	[46]
Virulence factor	*cylA*	Cytolysin toxin	[46]
Virulence factor	*efaA*	Cell surface antigen	[46]
Virulence factor	*esp*	Enterococcal surface protein, biofilm formation	[46,47]
Virulence factor	*gelE*	Gelatinase, biofilm formation	[46]
Virulence factor	*hyl*	Hyaluronidase, tissue invasion	[46]

Table S-4. Reference genomes for each VRE *Enterococcus faecium* clade

Clade	Reference name	NCBI RefSeq assembly
A1	AUS004	GCF_000250945.2
A2	F88	GCF_019175425.1
B	BIOPOP3-WT	GCF_012045505.1

Table S-5. List of competing methods and parameters for the genomic clustering sensitivity analysis.

Genomic Method	Parameters	Reference for parameters
Split-kmer analysis (SKA)	default	[12]
Split-kmer analysis (SKA)	optimized	[28]
Alignment-based methods	Not applicable	Not applicable

Section 5: Results

While the total number of samples collected from the four main sampling sources (patients, HCP hands, patient room environments, and shared environments) totaled 6848, there were additional samples that were collected for quality control. Overall, the total number of samples tested for microbiologic detection was 7000. These included controls and samples from the sampler hands. These went through identical microbiologic testing to ensure quality control and rule out laboratory contamination. None of the controls or sampler hand samples yielded VRE colonies, so they are not included in our analysis.

medRxiv preprint doi: https://doi.org/10.64898/2026.07.28.26359040; this version posted July 30, 2026. The copyright holder for this preprint (which was not certified by peer review) is the author/funder, who has granted medRxiv a license to display the preprint in perpetuity.
It is made available under a CC-BY 4.0 International license .

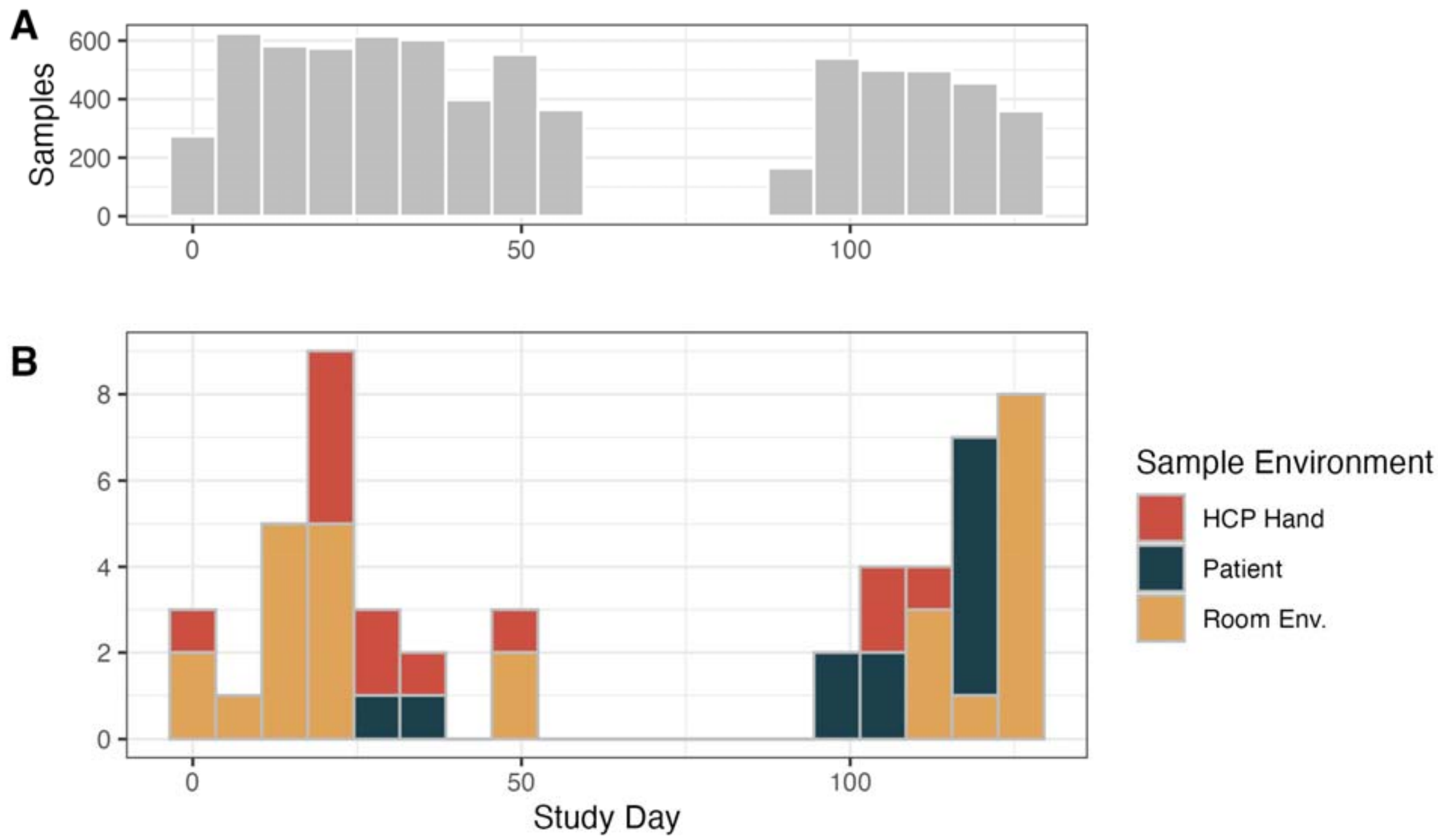

Figure S-1. Study timeline of sampling effort and vancomycin-resistant Enterococcus (VRE) detection. A) Histogram of the total number of samples collected each week, with hospital A samples collected days 1-56 and hospital B samples collected days 93-127. B) Histogram of all samples yielding one or more VRE isolate over time, colored by the sample environment. Since no VRE was isolated from the shared environmental surfaces, it is omitted from this histogram.

medRxiv preprint doi: https://doi.org/10.64898/2026.07.28.26359040; this version posted July 30, 2026. The copyright holder for this preprint (which was not certified by peer review) is the author/funder, who has granted medRxiv a license to display the preprint in perpetuity.
It is made available under a CC-BY 4.0 International license .

Table S-6. *Count of samples yielding at least one VRE isolates (Number of samples collected, %), by facility and sampling environment.*

	Healthcare facility		
**Sample environment**<br>Count (Collected, %)	**Hospital A**	**Hospital B**	**Total**
**Patient**			
Axilla	0 (369, 0.0%)	0 (157, 0.0%)	0 (526, 0.0%)
Groin	0 (306, 0.0%)	2 (129, 1.6%)	2 (435, 0.5%)
Perianal	2 (221, 0.9%)	7 (109, 6.4%)	9 (330, 2.7%)
**Patient Total**	**2 (896, 0.2%)**	**9 (395, 2.3%)**	**11 (1291, 0.9%)**
**Room Env.**			
Zone 1: Near Patient	7 (838, 0.8%)	3 (262, 1.1%)	10 (1100, 0.9%)
Zone 2: HCP Area	4 (236, 1.7%)	0 (262, 0.0%)	4 (498, 0.8%)
Zone 3: Toilet Area	1 (267, 0.4%)	3 (262, 1.1%)	4 (529, 0.8%)
Bedrail	0 (67, 0.0%)	0 (3, 0.0%)	0 (70, 0.0%)
Computer Cart	0 (61, 0.0%)	---	0 (61, 0.0%)
Door Handle	0 (61, 0.0%)	0 (3, 0.0%)	0 (64, 0.0%)
Table	0 (66, 0.0%)	0 (3, 0.0%)	0 (69, 0.0%)
Toilet	0 (61, 0.0%)	0 (3, 0.0%)	0 (64, 0.0%)
Sink	0 (12, 0.0%)	1 (184, 0.5%)	1 (196, 0.5%)
Computer Monitor	---	0 (3, 0.0%)	0 (3, 0.0%)
**Room Env. Total**	**12 (1669, 0.7%)**	**7 (985, 0.7%)**	**19 (2654, 0.7%)**
**HCP Hand**			
Glove	5 (953, 0.5%)	1 (268, 0.4%)	6 (1221, 0.5%)
Bare Hand	3 (772, 0.4%)	2 (388, 0.5%)	5 (1160, 0.4%)
One of each	0 (3, 0.0%)	0 (2, 0.0%)	0 (5, 0.0%)
Missing	0 (10, 0.0%)	0 (6, 0.0%)	0 (16, 0.0%)
**HCP Hand Total**	**8 (1738, 0.5%)**	**3 (664, 0.5%)**	**11 (2402, 0.5%)**
**Shared Env.**			
Shared Nursing	0 (95, 0.0%)	0 (35, 0.0%)	0 (130, 0.0%)
Shared Equipment	0 (52, 0.0%)	0 (35, 0.0%)	0 (87, 0.0%)
Supply cart	---	0 (284, 0.0%)	0 (284, 0.0%)
**Shared Env. Total**	**0 (147, 0.0%)**	**0 (354, 0.0%)**	**0 (501, 0.0%)**
**Overall**			
**Total**	**22 (4450, 0.5%)**	**19 (2398, 0.8%)**	**41 (6848, 0.6%)**

Figure S-2.

medRxiv preprint doi: https://doi.org/10.64898/2026.07.28.26359040; this version posted July 30, 2026. The copyright holder for this preprint (which was not certified by peer review) is the author/funder, who has granted medRxiv a license to display the preprint in perpetuity.
It is made available under a CC-BY 4.0 International license .

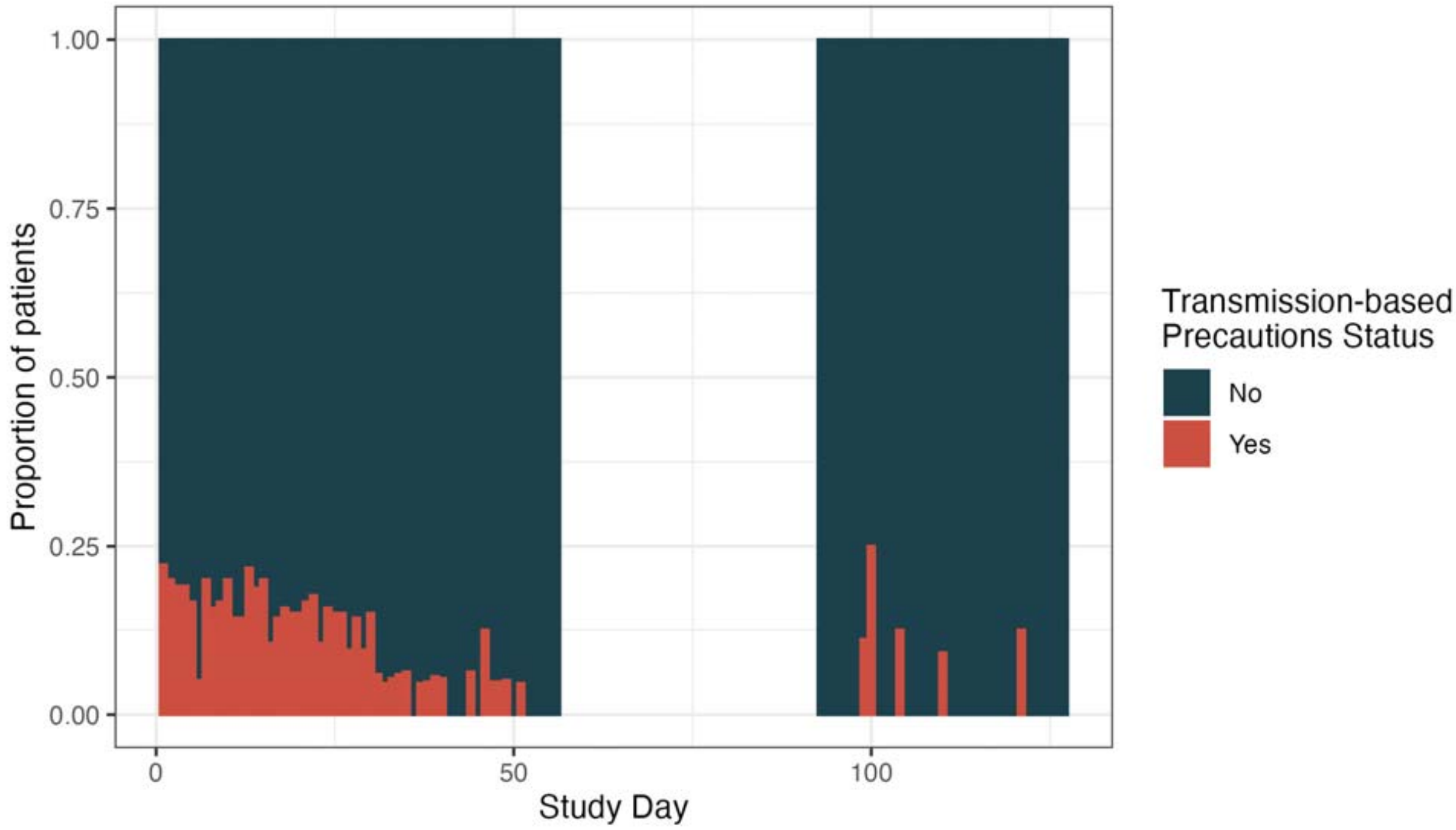

Figure S-2. Proportion of patients in hospital A's CV-ICU (study days 1-56) and hospital B's ICU (study days 93-127) on any type of transmission-based precautions.

Figure S-3.

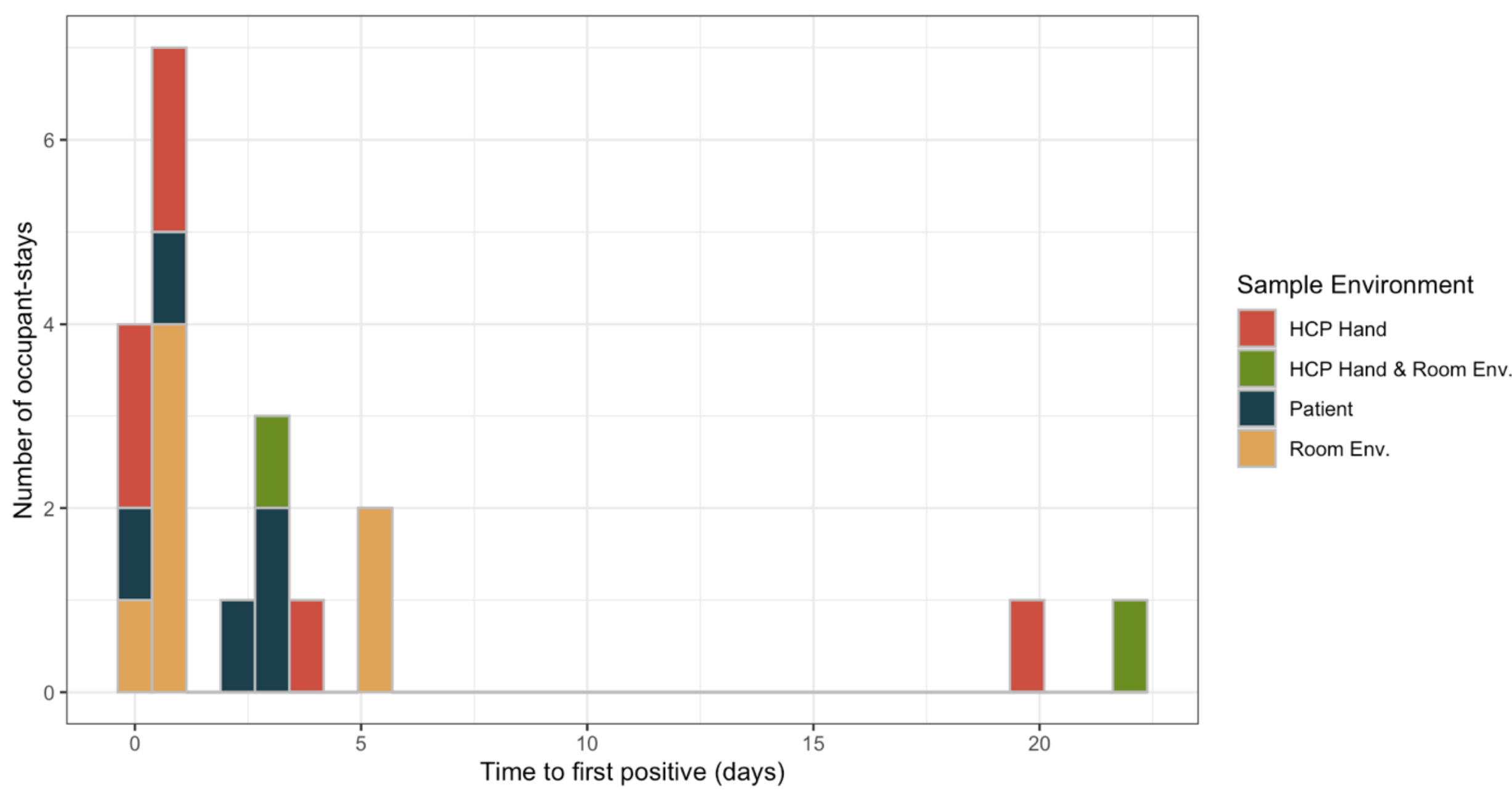

Figure S-3. Histogram of occupant-stays with VRE detection with the time (in days) between patient admission and VRE detection, with fill color representing the sample environment that first yielded VRE.

medRxiv preprint doi: https://doi.org/10.64898/2026.07.28.26359040; this version posted July 30, 2026. The copyright holder for this preprint
(which was not certified by peer review) is the author/funder, who has granted medRxiv a license to display the preprint in perpetuity.
It is made available under a CC-BY 4.0 International license .

Figure S-4

medRxiv preprint doi: https://doi.org/10.64898/2026.07.28.26359040; this version posted July 30, 2026. The copyright holder for this preprint (which was not certified by peer review) is the author/funder, who has granted medRxiv a license to display the preprint in perpetuity.
It is made available under a CC-BY 4.0 International license .

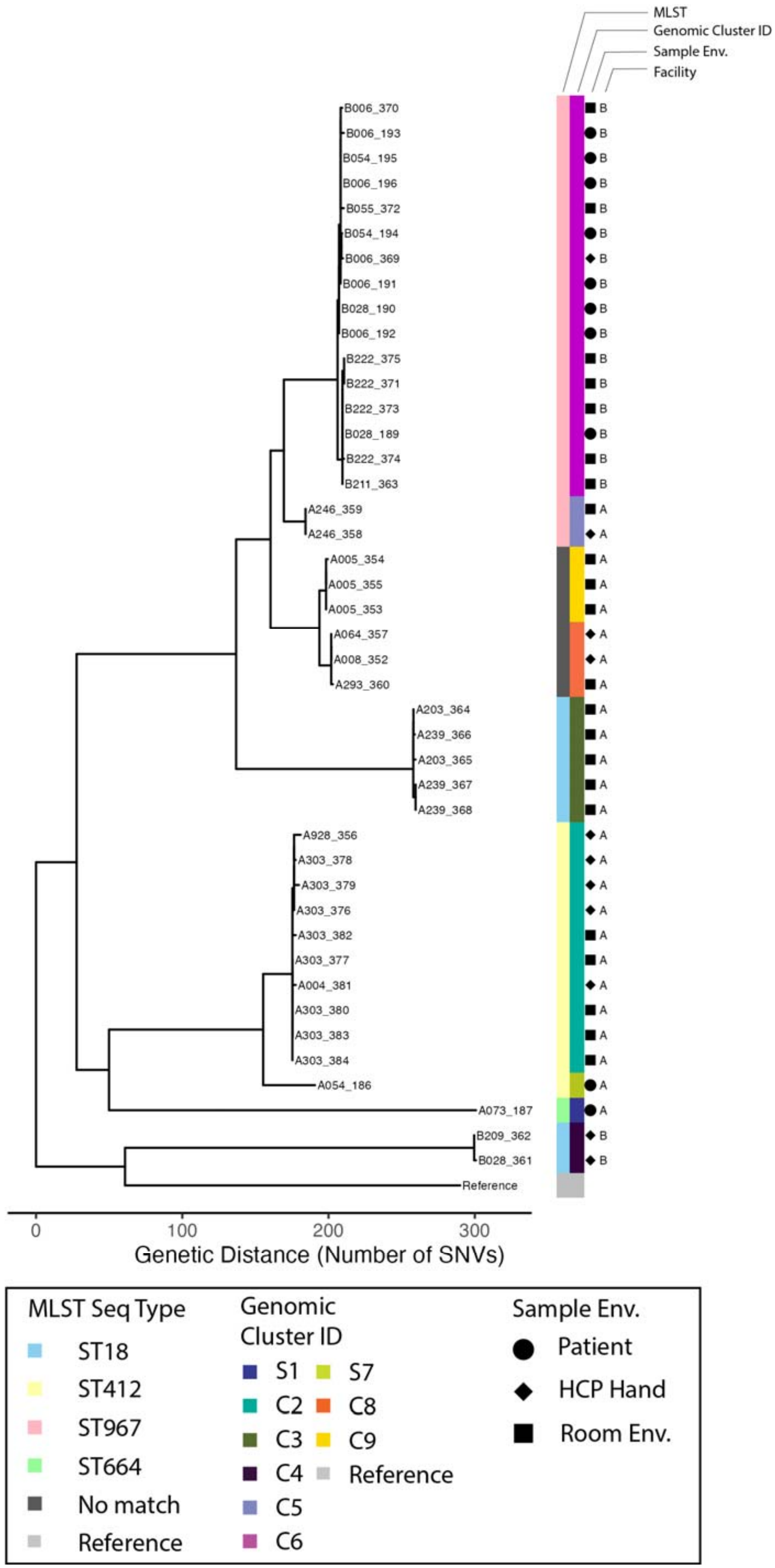

medRxiv preprint doi: https://doi.org/10.64898/2026.07.28.26359040; this version posted July 30, 2026. The copyright holder for this preprint (which was not certified by peer review) is the author/funder, who has granted medRxiv a license to display the preprint in perpetuity.
It is made available under a CC-BY 4.0 International license .

Figure S-4. Maximum likelihood phylogenetic tree of all vancomycin-resistant Enterococcus faecium (VREfm) isolates generated after alignment against the Clade A1 reference genome (AUS004) and recombinant region filtering. Tips are labeled with the occupant-stay identifier that their samples are affiliated with, followed by the whole genome sequence identifier (e.g., A303_384). Metadata associated with each tip include the multilocus sequence types (MLST) of each isolate when available, the unique Cluster ID as identified via our clustering algorithm, the sample environment, and the healthcare facility.

Figure S-5.

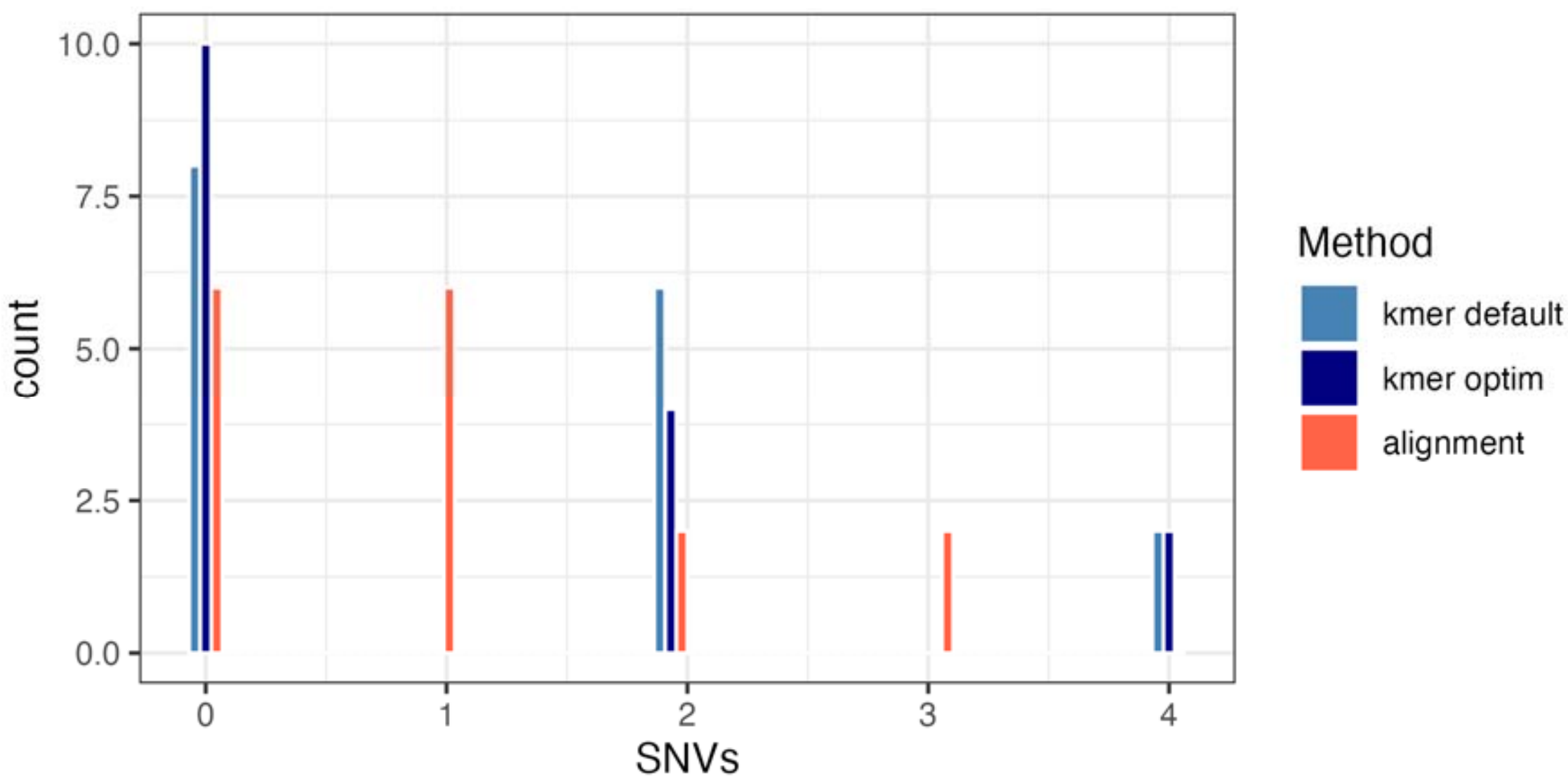

Figure S-5. Histogram of the pairwise SNV-distances calculated between all pairs of VRE isolates taken from the same patient, body site samples only. The pairwise SNVs calculated using each method are depicted by the color of the bar, with kmer-based methods using different parameters in blue, and the alignment based method in red.

medRxiv preprint doi: https://doi.org/10.64898/2026.07.28.26359040; this version posted July 30, 2026. The copyright holder for this preprint (which was not certified by peer review) is the author/funder, who has granted medRxiv a license to display the preprint in perpetuity.
It is made available under a CC-BY 4.0 International license .

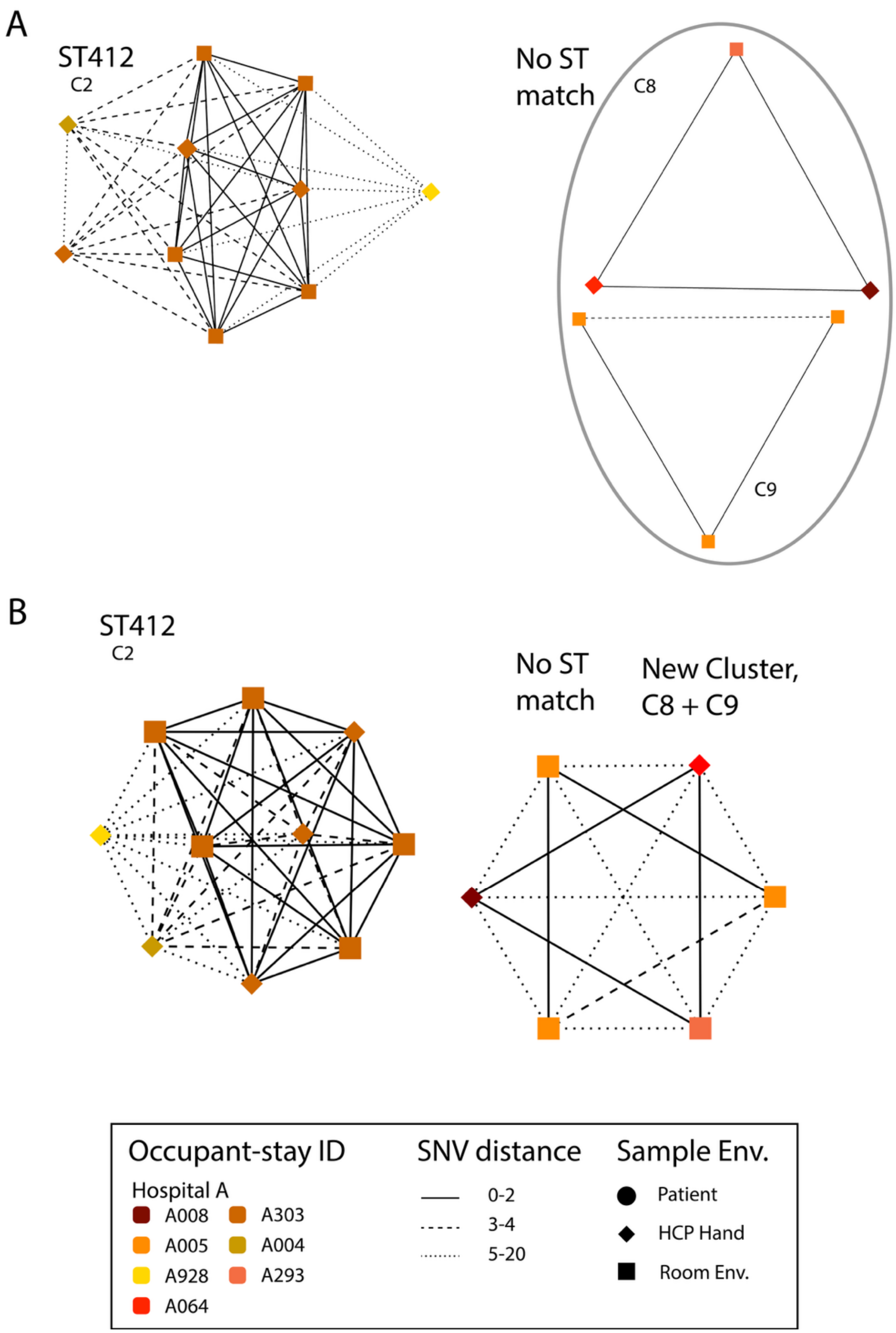

Figure S-6. Network plots of VREfm (vancomycin-resistant Enterococcus faecium) multi-isolate clusters from the single nucleotide variant (SNV) threshold sensitivity analysis. A) Network plots of VREfm clusters using the 7 SNV pairwise distance threshold for clustering links. B) Network plots of VREfm clusters using a 20 SNV pairwise distance threshold for clustering links. Nodes represent individual VREfm isolates, which are colored based on the occupant-stay that the sample was affiliated

medRxiv preprint doi: https://doi.org/10.64898/2026.07.28.26359040; this version posted July 30, 2026. The copyright holder for this preprint (which was not certified by peer review) is the author/funder, who has granted medRxiv a license to display the preprint in perpetuity.
It is made available under a CC-BY 4.0 International license .

with, and with shapes based on the sample environment. Edges that connect each node are weighted by the pairwise SNV distances. Only clusters that changed from the 7 to the 20 SNV thresholds are depicted. Clusters are labeled by their matching multi-locus sequence type. Sequence types with more than one cluster are circled in gray.
